# Protection of Permafrost Soils from Thawing by Increasing Herbivore Density

**DOI:** 10.1038/s41598-020-60938-y

**Published:** 2020-03-17

**Authors:** Christian Beer, Nikita Zimov, Johan Olofsson, Philipp Porada, Sergey Zimov

**Affiliations:** 1 Department of Environmental Science and Analytical Chemistry, Stockholm University, Stockholm, Sweden; 2 Bolin Centre for Climate Research, Stockholm University, Stockholm, Sweden; 3 Institute of Soil Science, Department of Earth Sciences, Faculty of Mathematics, Informatics and Natural Sciences, Universität Hamburg, Hamburg, Germany; 4 North-East Scientific Station, Pacific Institute for Geography, Far-East Branch, Russian Academy of Sciences, Cherskii, Russia; 5 Department of Ecology and Environmental Sciences, Umeå University, Umeå, Sweden; 6 Institute of Plant Science and Microbiology, Department of Biology, Faculty of Mathematics, Informatics and Natural Sciences, Universität Hamburg, Hamburg, Germany; 7 Center for Earth System Research and Sustainability, Universität Hamburg, Hamburg, Germany

**Keywords:** Carbon cycle, Cryospheric science

## Abstract

Climate change will cause a substantial future greenhouse gas release from warming and thawing permafrost-affected soils to the atmosphere enabling a positive feedback mechanism. Increasing the population density of big herbivores in northern high-latitude ecosystems will increase snow density and hence decrease the insulation strength of snow during winter. As a consequence, theoretically 80% of current permafrost-affected soils (<10 m) is projected to remain until 2100 even when assuming a strong warming using the Representative Concentration Pathway 8.5. Importantly, permafrost temperature is estimated to remain below −4 °C on average after increasing herbivore population density. Such ecosystem management practices would be therefore theoretically an important additional climate change mitigation strategy. Our results also highlight the importance of new field experiments and observations, and the integration of fauna dynamics into complex Earth System models, in order to reliably project future ecosystem functions and climate.

## Introduction

Human societies currently emit more than 10 Gt carbon (Gt C) every year into the atmosphere in form of carbon dioxide^1^ which is a long-lived greenhouse gas (GHG). It has been estimated on the basis of Earth System Model (ESM) projections that approximately additional 150–330 Gt C could still be emitted in total until 2080 followed by negative emissions in order to keep global warming below 1.5–2.0 °C relative to the pre-industrial era^2,3^. However, additional to any future *anthropogenic* GHG emissions, there is a risk of additional, climate change-induced GHG releases from geological reservoirs by *natural* processes which have not been taken into consideration in ESM projections so far. A prominent reservoir is high-latitude permafrost-affected soils (gelisols) containing organic matter that has been accumulated since the Pleistocene. About 800 Gt C have been estimated to be stored in the perennially frozen part of these soils^4^ which has been always frozen since thousands of years, thereby preserved from microbial decomposition. With the projected amplified increase in surface air temperature in the Arctic until 2100^5^ the active layer, which is the uppermost part of the soil thawing each summer and freezing back in autumn, will warm and also deepen, and thereby thawing currently perennially frozen ground^6–8^. Consequently, more organic matter will be decomposed by microbes, and additional 11–143 Gt C are projected to be released to the atmosphere as CO_2_ until 2100^9,10^, but potentially 100 to 600 Gt C until 2300^8,10,11^. Such a substantial future carbon release will reduce the anthropogenic emission budget and will form a positive feedback mechanism^12–14^ with an additional warming of about 1.4 °C until 2100^9^. Therefore, managing ecosystems in a way that geological carbon reservoirs - and in particular perennially frozen soil organic matter - are conserved may represent an important climate change mitigation strategy.

One particular possible mitigation strategy is the additional introduction and management of herbivores, such as reindeer, horses, bison, etc. in contemporary northern high-latitude ecosystems. In the late Pleistocene the mammoth steppe ecosystem consisted of numerous herbivores of about 10 ton per km^2^ biomass^15^ and occupied most of Northern Eurasia. Since the beginning of the Holocene, big mammals disappeared and the mammoth steppe vanished. Today, only reindeer is found^16^ with a density below 10 individuals per km^2^ in most of the Arctic^17^. This can, however, be changed since most populations of large herbivores like reindeer and muskoxen are directly managed by humans, either by hunting or management^18^. The herbivore community can also be manipulated even more by reintroducing lost components of the Arctic herbivore assembly. In a huge and long-term experiment called Pleistocene Park, a 2000 hectare area in the Kolyma river lowland, Russian Far East has been fenced in 1996^19^. Then, different herbivores have been introduced into this park in order to study their effect on plant biodiversity, vegetation productivity, and soil temperature regime. Winter grazing and movements by the animals compact snow, thereby substantially decreasing the thermal insulation efficiency of snow. This allows much colder freezing of soil in winter, hence colder overall mean annual soil temperature. The hypothesis is that this cooling effect may prevent permafrost from thawing or at least postpone the degradation^15^. However, to test this hypothesis, a quantitative assessment is needed on the long-term effect of increasing snow compaction until 2100 under climate change. Would a high potential increase in the population density of large herbivores preserve permafrost temperature and gelisol extent until the end of the century? Or, would the increasing air temperature forcing anyhow dominate over the reduced soil insulation effect, and thus lead to a positive permafrost carbon-climate feedback mechanism?

In order to address these questions, here we use snow depth and soil temperature observations in concert with the land surface model JSBACH that is state-of-the-art in terms of process representations for cold regions^20,21^. This model has been extensively evaluated at site level, regional scale, and global scale^20–25^. Model results central for this study (snow depth, land surface temperature, and mean annual ground temperature) as well as the insulation efficiency of snow are additionally evaluated against observations and discussed in light of other global model results in the Supplementary Information. JSBACH is a typical land surface model that solves the vertical heat conduction equation for five snow layers, one bryophyte/lichens layer, and seven soil layers using an implicit numerical scheme and explicitly considering the latent heat of fusion during phase change^20^. Grid cell size of the forcing data in this study is 0.5 degree. The height of the thermal soil layers increase with depth from 6 cm to 30 m reaching 53 m in total^21^. Soil depth until bedrock, which is the maximum depth of hydrological soil layers considered in the model, is additionally prescribed^25^ and usually ranges between 0.5–4 m in northern permafrost regions^26,27^. The model assumes homogeneous soil conditions within a grid cell, and thermal and hydrological parameters have been derived using pedo-transfer functions^20^ based on soil texture type information from the Harmonized World Soil Database at 1 km horizontal resolution^28^. In this study, JSBACH runs decoupled from the atmospheric model and instead has been forced by harmonized climate data during 1901–2100. The bias-correction method is explained in detail in refs. ^29,30^. WATCH forcing data and ECMWF ERA-Interim data have been used for the historical period 1901-2010. For the period 2011–2100, CMIP5 output of the Max-Planck-Institute Earth System Model^31^ has been applied following the Representative Concentration Pathway 8.5 (RCP8.5). We concentrate here on this scenario as it shows the strongest warming signal and therefore is best suited to investigate conservatively the potential of big herbivores to save permafrost from thawing. A typical model experiment includes several hundreds of years spin-up of physical state variables, such as water and ice content, and soil temperature, in concert with 10000 years spin-up of carbon pools using an average pre-industrial climate and atmospheric CO_2_ content. In addition to the control model experiment (CNTL), a modified version of JSBACH (PlPark experiment) has been run during 2020–2100. In this experiment, the snow compaction rate and the maximum snow density were set at higher values, emulating the effect of winter grazing activities of herbivores, based on observations from Northern Sweden and the Kolyma river lowland in Siberia (methods sections).

## Results

### Observed reindeer effects on snow depth

**Figure 1:**
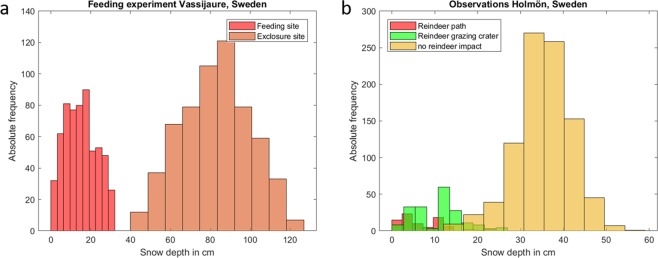
Histograms of snow depth observations at two locations in Sweden with/without reindeer impact.

Systematic snow depth measurements at two sites in Northern Sweden document clearly the effect of reindeer on snow depth. At the feeding site close to Vassijaure, Sweden, snow depth has been measured to be 15 cm on average, a reduction of 82% compared to the control site (83 cm on average) under the same meteorological and environmental conditions. The respective two histograms even do not overlap (Fig. 1a). Interestingly, the animals also reduce the standard deviation by 50% from 17 cm to 8 cm. At Holmön, Sweden, snow depth has been observed along with respect to reindeer impact: no impact, reindeer path, and grazing crater. Here, the strong impact of reindeer on snow depth has been additionally confirmed. On Holmön, mean snow depth of reindeer-impacted areas (7.8 cm at reindeer paths, 11.2 cm at grazing craters) is 73% reduced compared to the snow depth of unaffected areas (35 cm) on average (Fig. 1b). The areal coverage of reindeer-affected snow has been estimated at 23%. That translates into an average snow depth of 29 cm instead of 35 cm, a reduction by reindeer of 17%. In the Pleistocene Park in Cherskii, Russia, a herbivore density of 114 individuals per km^2^ led to an overall average reduction of snow depth by 50%

### Observed effects of mammals on soil temperature

**Figure 2:**
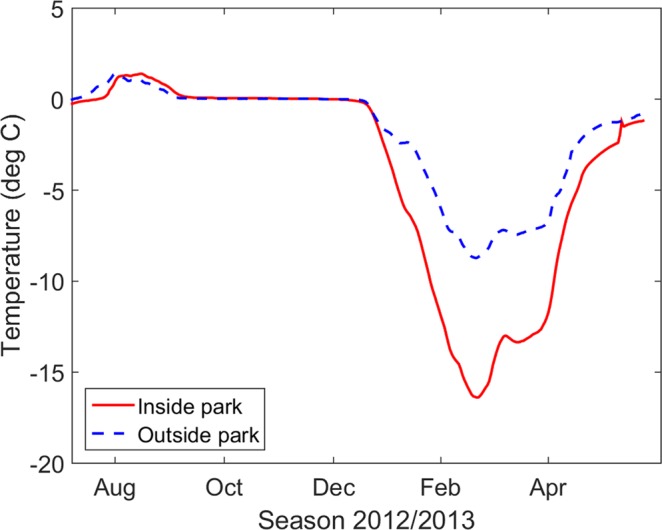
Comparison of soil temperature observations (°C) at 90 cm depth inside and outside the Pleistocene Park, Kolyma river lowland, Russian Far East during one year. The mean annual difference is −1.9 °C.

Soil temperature observations inside and outside the Pleistocene Park close to Cherskii, Russian Far East, demonstrate the general effect of increasing the mammal population on soil temperature (Fig. 2). While summer and autumn temperature at 90 cm soil depth are similar at both sites, the effect of different snow depth and density on soil temperature is clearly visible during winter and spring (Fig. 2) with a mean annual difference of 1.9 °C between the two sites.

### Simulation results of snow properties

**Figure 3:**
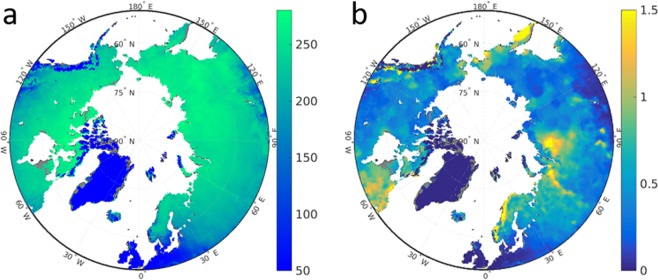
JSBACH CNTL experiment mean snow properties during 1990–2010. (**a**) DJF snow density (kg m^−3^) and (**b**) annual maximum snow depth (m).

The CNTL model simulates December-January-February (DJF) averages of snow density during 1990–2009 of 200–260 kg m^−3^ (Fig. 3a). The resulting maximum monthly snow depth varies depending on the region between 0.2 and 1.5 m (Fig. 3b). These results are generally in agreement with observations and other modeling studies (Supplementary Information). Importantly, the model also represents correctly the thermal diffusion through snow, hence the strength of the insulation of soil by the snow (Supplementary Information Fig. S3).

**Figure 4:**
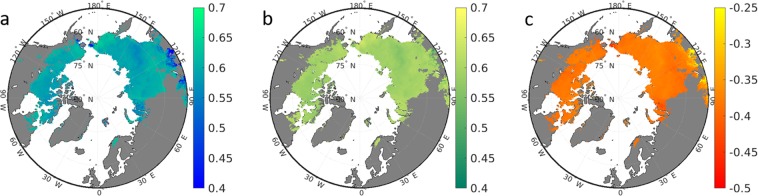
Simulated effects of big mammals on snow properties. Shown are relative differences (−) between PlPark and CNTL model experiments of December-January-February averages during 2090–2099 of (**a**) snow density, (**b**) snow thermal diffusivity, and (**c**) snow depth. Grey color denotes land outside the historical (1990–2009) JSBACH estimate of permafrost zone.

In the PlPark model experiment both the higher compaction rate and the higher possible maximum snow density lead to an overall increase in the DJF snow density of 50–70% compared to the CNTL model run (Fig. 4a). This increase in snow density has a direct effect on the insulation efficiency of snow via thermal properties, and an indirect effect via the depth of this insulating layer. First, the higher snow density leads to also 50–65% higher snow thermal diffusivity (Fig. 4b), i.e. the heat diffusion between soil and atmosphere is much more effective. While the amount of winter precipitation per m^2^ ground is exactly the same in both simulations, the higher snow density additionally leads to a reduction of snow depth of mostly 30–45% in the PlPark experiment (Fig. 4c). Taken together, trampling down the snow by large herbivores in winter and the respective increase in snow density leads to a better connection of the atmosphere to the soil both due to a higher thermal diffusivity and due to a reduction of the insulating layer height.

### Future permafrost temperature and permafrost area extend

**Figure 5:**
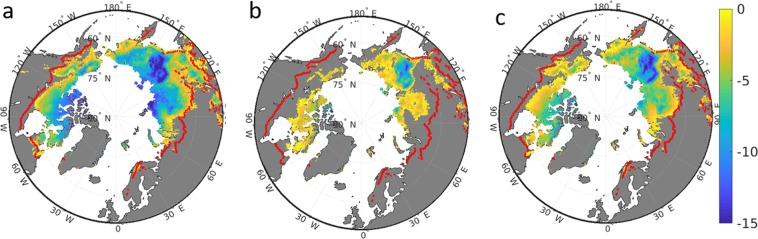
Spatial details of permafrost temperature (°C, 4–10 m average). (**a**) CNTL experiment during 1990–2009. (**b**) CNTL experiment during 2090–2099. (**c**) PlPark experiment during 2090–2099. Grey color denotes land outside the JSBACH estimate of permafrost zone. The red line represents the observation-based^32^ contemporary southern boundary of continuous and discontinuous permafrost.

Permafrost temperature during 1990-2009 is simulated to range between −10 °C in the High Arctic to 0 °C at the southern fringe (Fig. 5a). The areal average is −6.7 °C (Table 1). These results are in good agreement with borehole observations (Supplementary Information Fig. S5). An exception is East Siberia where simulation results can be as low as −15 °C . Figure 5a also shows that the southern border of what we define as permafrost area based on model results (methods) agree well with the observation-based estimate of the southern boundary of the continuous and discontinuous permafrost zone. Assuming the RCP8.5 scenario, the land surface model suggests a reduction by about half the contemporary permafrost area until the end of the century (Table 1, Fig. 5b), and the remaining permafrost is suggested to be on average 3.8 °C warmer than today (Table 1). Usually, permafrost temperature in this scenario is estimated to be just a few degrees Celcius below the freezing point, again with the exception of East Siberia (Fig. 5b).

**Table 1:** CNTL and PlPark model experiment results of permafrost extent (Mha) and mean annual ground temperature (MAGT, 4–10 m average, in °C).

Period	CNTL		PlPark	
	Area (Mha)	Temperature (°C)	Area (Mha)	MAGT (°C)
1990–2009	1209	− 6.7	1209	− 6.7
2090–2099	578	− 2.9	976	− 4.6
Difference	− 631	3.8	− 233	2.1

In contrast, the PlPark model experiment suggests a colder permafrost soil (2.1 °C difference on average, Table 1) at the end of the century as a consequence of the effects of higher herbivore density on snow density. In large areas, permafrost temperature is now simulated to stay below −5 °C even under the strong atmosphere warming scenario RCP8.5. The reduction of permafrost area is estimated to be only 233 Mha in this experiment instead of 631 Mha in the CNTL model experiment (Table 1, Fig. 5c).

### Parameter sensitivity study

In the PlPark experiment, snow and moss parameters have been adjusted to mimic the effects of herbivores on these temperature insulators. To study the relative importance of snow versus moss related parameters, a systematic parameter sensitivity study has been performed. Table 2 shows a high sensitivity of snow properties to the snow compaction rate constant and the maximum density of snow. As a consequence, permafrost temperature is also highly negatively correlated with snow parameters (−0.7 and −0.9). A higher turnover rate constant of mosses also leads to a reduction in moss cover, the correlation coefficient of −0.56, however, is quite moderate and point to other important environmental factors controlling moss cover. The overall sensitivity of permafrost temperature and permafrost extent to the moss turnover rate constant is negligible but highly sensitive to snow parameters (Table 2).

**Table 2:** Partial correlation coefficients between state variables (rows) averaged during 2090–2099 and model parameters (columns) used in a parameter sensitivity study.

	snow compaction	snow maximum	moss
	rate constant	density	turnover rate
Snow density	0.78	0.96	0.31
Snow diffusivity	0.75	0.96	0.31
Snow depth	− 0.76	− 0.96	− 0.29
Moss cover	0.32	0.42	− 0.56
Permafrost temperature	− 0.71	− 0.9	− 0.06
Permafrost area	0.66	0.87	− 0.02

**Figure 6:**
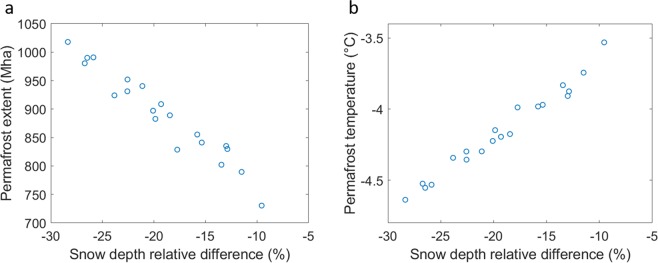
Sensitivity study mean areal results of permafrost extent (**a**) and permafrost temperature (**b**) as a function of percent snow depth difference to the CNTL model run for the period 2090–2099.

As a site-effect, the parameter sensitivity study also provides 20 more realizations of snow depth reduction and resulting permafrost extent and temperature in between the two extreme experiments CNTL and PlPark. Figure 6 shows model results of permafrost extent or temperature during 2090–2099 for simulations with 10 to 30% reduction in snow depth on average.

## Discussion

In response to a warming of the Arctic, observations already show permafrost warming by about 0.2–2 °C during the past decades^33–35^. The JSBACH model agrees with a conservative global-scale increase in permafrost temperature of 0.7 °C during 1980-2010 (Supplementary Information Fig. S6). Assuming the Representative Concentration Pathway 8.5, permafrost temperature is projected to further increase by 2–10 °C until the end of the century (Fig. 5a,b) in accordance with other model simulations^6,7^. As a result, mean annual ground temperature will be above the freezing point in many contemporary permafrost regions, which translates into an enormous loss of gelisol extent in line with other model results^8,9^. Due to the additionally increasing permafrost temperature (Fig. 5b), most of the remaining permafrost soils in 2100 will even further thaw until 2300 assuming the RCP8.5^8^.

Our question was: If we were able to initiate and maintain a high density of herbivores comparable to the situation in the Pleistocene Park in Cherskii, Russia, could that change in herbivore density prevent contemporary permafrost from thawing? Or would atmospheric warming still be the dominant forcing overriding any snow insulation effect? The respective PlPark model experiment, with snow depth difference to the CNTL model run of about 30 to 45% (Fig. 4c) comparable to the 50% snow depth reduction in the Pleistocene Park, shows an approximately 44% reduced subsoil warming and a 37% reduced loss in permafrost area compared to the CNTL run (Table 1). The projected future permafrost temperature of −4.6 °C on average in the PlPark model experiment is 1.7 °C colder than the CNTL model estimate (−2.9 °C, Table 1), which is comparable to the difference of 1.9 °C observed under contemporary climate conditions in the field experiment in the Kolyma river lowland (Fig. 2). In the PlPark model experiment, the still cold permafrost below −5 °C may also prevent a further fast thawing of huge areas during the upcoming centuries even under the RCP8.5^8^. We concentrate on RCP8.5 in this study because this scenario represents the upper end of assumed anthropogenic CO_2_ emissions with the strongest atmospheric warming in the Arctic. Preventing permafrost soil conditions under this scenario demonstrates the overall strength of the approach of increasing herbivore density and can be even more effective and important under mitigation RCPs, such as RCP4.5 or RCP2.6.

The herbivore density in the site close to Vassijaure railway station (483 individuals per km^2^) is very high and possibly not reachable at a pan-Arctic scale. This data is just used to demonstrate the upper range of effects of reindeer on snow depth. The density in the Pleistocene Park is with 114 individuals per km^2^ about 20 times higher than the average current density at a pan-Arctic scale of 5 individuals per km^2^^17^. Still, this experiment demonstrates in our view a theoretical upper end of possible herbivore density in tundra ecosystems, comparable with the situation during the late Pleistocene^15^. However, two important open questions remain: (1) What is the snow depth-herbivore density relationship at a landscape scale? (2) Which herbivore density is reachable and useful? With the few available data in hand we are not able to fully address these open questions. Our data from Holmön in Sweden shows that with a density of 15 individuals per km^2^, snow depth is reduced by 17% at a landscape scale. The sensitivity study with varying snow parameters now shows a remaining permafrost extent of 850 Mha at the end of the century for also such medium levels of snow depth reduction (Fig. 6a).

Introducing a large amount of big mammals into tundra and forest tundra ecosystems will also have other consequences for ecosystem functions, such as enhancing of primary productivity or nutrient cycling while at the same time reducing shrub and tree cover^15^. The resulting enhancement of both carbon dioxide uptake and surface albedo led to an additional negative feedback to global warming. However, higher grazing activities may also disturb near-surface vegetation such as lichens and bryophytes, thereby reducing their insulation efficiency in summer and leading to soil warming. Since our land surface model version includes a process-based representation of lichens and bryophytes^21^, we could explicitly account for such effects on soil temperature by doubling the turnover rate constant of mosses. The sensitivity study results suggest an overall negligible effect of moss turnover rate on permafrost temperature and illustrate the importance of snow properties (Table 2). However, such first model experiments do not take into consideration all interactions between ecological and physical processes. For instance, changing vegetation type and cover by herbivores will impact surface albedo and evapotranspiration. Therefore, our results demonstrate the need for further research on the effects of big herbivores on land-atmosphere interactions and on integrating fauna dynamics into complex Earth System Models.

Global-scale numerical simulation experiments demonstrated that the introduction of big herbivores into tundra ecosystems can prevent 37% of permafrost soils from thawing across the entire Arctic, such that 80% of permafrost soils with an average permafrost temperature below −4 °C will remain in 2100. Therefore, more efforts are needed to explore the implementation of such unconventional management practices as an effective climate change mitigation concept in addition to traditional emission reduction strategies. Our results suggest that the integration of fauna dynamics and ecological functions into complex Earth System models may be a crucial step towards a more realistic representation of ecosystem functions and more reliable projections of future climate. The assumption behind these estimates is that herbivore density can be on pan-Arctic scale as high as in the Pleistocene Park experiment in Cherskii, Russia. However, the sensitivity study shows that considerable less herbivore density and hence less snow depth reduction will also have a high potential to prevent permafrost from thawing. Hence, our study demonstrates the need of much more detailed field studies and experiments about the effect of herbivores, such as reindeer on snow depth at a landscape scale.

## Methods

### Snow depth observations, Northern Sweden

The effect of reindeer on snow depth has been measured in two study sites. The first is a large enclosure used for winter feeding of Norwegian reindeer (Gielas reindeer herding districts) in Northernmost Sweden, close to the Vassijaure railway station (68${}^{\circ }2{5}^{{\prime} }46.{2}^{{\prime\prime} }$N,$1{8}^{\circ }2{0}^{{\prime} }47.{3}^{{\prime\prime} }$E). Inside the enclosure, 700 reindeer were kept for 28 days in a 12 ha large enclosure during the winter 2017, corresponding to a density of 483 reindeer per km^2^ during that period. Snow depth was measured at 16 April 2017, a couple of weeks after the reindeer had left the enclosure. Snow depth was measured with an avalanche probe every 10 cm along six transects inside the enclosure and six transects outside the enclosure (undisturbed by reindeer). The transects were paired in sites with similar topography to avoid confounding factors, and the difference inside and outside the enclosures should thus relate to the presence of reindeer only. The second study site is the Holmön archipelago (63${}^{\circ }43.159{\prime}$N,20°$55.078{\prime}$E). The islands has occasionally been used as winter grazing area by Rans reindeer herding district. Between October 2015 and April 2016 about 700 reindeer were moved to the Holmön archipelago, which corresponds to a density of 15 reindeer per km^2^. Here, snow depth was measured at 17 March 2016 every 10 cm along twelve 10 m long transects with the same methods as in the first study site. Each point along the transect was also characterized as no impact by reindeer, trampling, or feeding crater. The densities in both these study areas was much higher than the average densities of reindeer presently found in the Arctic of 0 to 10 reindeer per km^2^^17^. However, since reindeer are migratory and move in large herds, extreme densities corresponding to the enclosure is sometimes found for a few weeks also under natural settings, and the Holmön island is a natural use of winter resources and corresponds to densities often found in an area a year that is used for winter grazing.

### Pleistocene park experiment, cherskii, russia

Pleistocene Park is the scientific experiment on reconstruction of highly productive steppe ecosystems in the Arctic^19^. The experiment is conducted in the Kolyma river lowland (68.51°N,161.50°E). It is a 2000 hectare area divided into subsections by game fences. Typical northern vegetation types- tussock grassland, willow shrubs and larch forest, originally represented the landscape. Starting 1996, different herbivores were introduced to the Park. Some species are native to the region, some used to inhabit the area in the Pleistocene. These are reindeer, Yakutian horses, moose, musk ox, European bison, yaks, cold adapted sheep. Density of herbivores is artificially kept above the feeding capacity of the pastures (by providing extra forage). This allows grasses and herbs to out-compete modern low productive vegetation and gradually increase productivity of the territory. To test the effect of winter grazing on snow cover and permafrost temperature, in 2011 soil temperature sensors in the year-round grassland pasture within the park and in the similar landscape but without grazing, 10 km outside of the park, have been installed. Temperature sensors were installed within the active layer on the depths of 10, 25, 50 and 90 cm. Nonstop temperature measurements were obtained from July 2011 to May 2013 for the non-grazed site and from July 2012 to July 2013 for the grazed site.

### Land surface model

In this study we use a version of the land surface scheme JSBACH^22,36,37^ that has recently been advanced by several processes which are particularly important in cold regions, including coupling of soil hydrology and vertical heat conduction via latent heat of fusion^20^ and the effects of ice and water content on soil thermal properties^20^, as well as a new dynamic snow model for soil insulation^21,25^. The version used here in particular also includes a dynamic biogeochemical model of lichens and bryophytes^21^ which simulates both the extent of lichens and bryophytes and their impact on the vertical heat conduction^21,25^. In total, five snow layers, one bryophyte/lichens layer, and seven soil layers are used in an implicit numerical scheme to solve the heat conduction equation with phase change^21,23^. Depth of thermal and hydrological layers increase from 6 cm at the surface to 30 m for the bottom layer. In sum, these layers reach a depth of 53 m ensuring no temperature amplitude in the last layer. However, the hydrological layers are restricted to the depth until bedrock, which typically range between 0.5 and 4 m in northern permafrost regions at the landscape scale. In this study, this information is based on ref. ^26^ as used in ref. ^27^. Horizontal resolution of model results is due to the resolution of forcing data (see below). Climate and soil datasets with a grid cell size of 0.5 degree are applied. Four dominant land cover classes are considered in each of these grid cells^36^. The coverage of these tiles has been estimated by combining several global land cover maps^20^. JSBACH interpolates daily climate forcing data to half-hourly values which is the internal time step of the model. More details on the model version used can be found in refs. ^20,21,25^. This model version has been intensively evaluated in terms of cold regions physical processes at site level and pan-Arctic scale^20,21,24,25^. Additional evaluation plots can be found in the supplemental information.

The advanced snow module of JSBACH represents a *dynamic* snow density (*ρ*_*snow*_. It is initialized with a minimum value of *ρ*_*min*_ = 50 *kg* *m*^−3^. The compaction effect is included as a function of time^38^ with a compaction rate *c* = −0.002 and a maximum density (*ρ*_*max*_ = 300 *kg* *m*^−3^),

1 $${\rho }_{snow}^{t+1}=\left({\rho }_{snow}^{t}-{\rho }_{max}\right)\exp \frac{c\cdot \Delta t}{3600}+{\rho }_{max}$$

where Δ*t* is the timestep length of model simulation in seconds. Snow density is calculated as a weighted mean of fresh snow with snow density $\left({\rho }_{min}\right)$ and the previous timestep’s value. Snow density controls snow heat capacity and conductivity^25^ and snow depth. Both, snow thermal properties and snow depth impact the insulation characteristic of snow and hence soil temperature. A higher snow density (e.g. due to herbivore grazing in winter) leads to a higher heat conductivity hence stronger heat flux in winter (soil cooling), and a higher snow density also leads to a lower snow depth hence closer atmosphere-soil coupling in winter (soil cooling).

### Climate forcing data

The JSBACH model driver estimates half-hourly climate forcing data using daily data of maximum and minimum air temperature, daily precipitation, short-wave and long-wave radiation, specific humidity and surface pressure. We are using global data at 0.5 degree spatial resolution. The historical data from 1901-1978 came from the WATCH forcing dataset^39^ at the same resolution. For the period 1979-2010, ERA-Interim reanalysis data have been downloaded from ECMWF also at 0.5 degree grid cell size^40^. This dataset has been bias-corrected against the WATCH forcing data. Climate data for future projections (2011–2100) have been obtained from the CMIP5 output of the Max-Planck-Institute Earth System Model^31^ following the RCP8.5. The original grid cell size of this dataset of 1.875 degree has been automatically improved to 0.5 degree by the bias-correction approach, which, in principle, projects the anomalies of the MPI-ESM time series onto a long term average of the reference dataset. For details about the bias-correction method please see refs. ^29,30^. Historical and future atmospheric CO_2_ concentration was prescribed following the CMIP5 protocol^41^.

Grid cells are divided into four tiles according to the four most dominant vascular plant functional types of this grid cell^20^. This vascular vegetation coverage is assumed to stay constant over the time of simulation. Hydrological parameters have been assigned to each soil texture class^42^ according to the percentage of sand, silt and clay at 1 km spatial resolution as indicated by the Harmonized World Soil Database^28^. Thermal parameters have been estimated^20^ at a 1 km spatial resolution. Then, averages of 0.5° grid cells have been calculated. An updated map of soil depth down to the bedrock^26,27^ has been applied.

### Simulation protocol for CNTL and PlPark model experiments

Climate forcing during the spin-up time consisted of randomly selected years during 1901-1930 from the climate dataset described above. Pre-industrial atmospheric carbon dioxide concentration was assumed to be 284 ppmv. First, JSBACH has been run for 30 years without enabling the freezing scheme in order to bring soil water reservoirs in a first equilibrium with climate. Then, JSBACH was again running 170 more years with the same forcing data but with enabling the freezing scheme in order to equilibrate soil temperature, soil water and ice content as well as lichen and bryophyte cover according to pre-industrial conditions. Afterwards, the spin-up model for the carbon pools was run for 10 k-years using JSBACH output from the last 30 years of the pre-industrial spin-up run. JSBACH was then run from 1850 to 1900 using again the random spin-up climate from the period 1901–1930 but using transient atmospheric CO_2_ concentration, followed by a fully transient run until 2100 using the climate data described above and dynamic atmospheric CO_2_ content following the RCP8.5^41^.

JSBACH has been additionally run during 2021–2099 starting from the CNTL experiment state variable conditions in 2020 but with

- increased snow compaction rate (*c* = −0.003 *h*^−1^),
- increased maximum snow density (*ρ*_*max*_ = 450 *kg* *m*^−3^), and
- a doubling of the moss turnover rate constant.

This simulation emulates an increase of big mammals, such as horses, reindeer, bison etc. in 2020 leading to increasing snow density, comparable to the Pleistocene Park experiment in Cherskii, Russian Far East. The experiment is therefore called PlPark. All other parameters and forcing data remain the same as in the CNTL simulation.

### Definitions, data analyses and plotting

Mean annual ground temperature (MAGT) is calculated as an average of soil temperature in 4 to 10 m depth. In this depth the temperature is fluctuating only marginally. Permafrost temperature is defined as MAGT in permafrost areas. The model results are then spatially plotted and analysed over the northern permafrost area, which is defined by 1990–2009 or 2090–2099 permafrost temperature lower than zero degrees Celcius. All land outside this permafrost zone is marked in grey color. The area of all permafrost-defined grid cells is summed as a pan-Arctic estimate. Grid cells that are treated as permanent glaciers in the model are excluded from this analysis.

### Parameter sensitivity study

In order to study the impact of snow and moss parameters on permafrost temperature and extent, the three parameters maximum snow density, snow compaction rate, and moss turnover rate have been varied simultaneously using a Latin hypercube sampling design resulting in 20 additional model runs. Ranges of possible parameter values are as follows: maximum snow density: 350–450 *kg* *m*^−3^, snow compaction rate: 0.002–0.003 *h*^−1^, and moss turnover rate: 0.01–0.02 *a*^−1^.

## Supplementary information

Supplementary Information.

## Data Availability

The climatic fields used in this study as forcing data for the JSBACH model are available upon registration under the following link (the tag "Geocarbon” has to be selected): https://www.bgc-jena.mpg.de/geodb/projects/Home.php. JSBACH output data which are presented as maps in this study are available as netCDF files from the authors on request. Snow depth and soil temperature observations are available as referenced in the supplementary information. The land surface model JSBACH used in this study is intellectual property of the Max Planck Society for the Advancement of Science, Germany. The JSBACH source code is distributed under the Software License Agreement of the Max Planck Institute for Meteorology, and it can be accessed on personal request. The steps to gain access are explained under the following link: http://www.mpimet.mpg.de/en/science/models/license/.

## References

[1] Le Quéré C (2018). Global carbon budget 2017. Earth Syst. Sci. Data.

[2] Millar RJ (2017). Emission budgets and pathways consistent with limiting warming to 1.5 °C. Nat. Geosci..

[3] Goodwin P (2018). Pathways to 1.5 °C and 2 °C warming based on observational and geological constraints. Nat. Geosci..

[4] Hugelius G (2014). Estimated stocks of circumpolar permafrost carbon with quantified uncertainty ranges and identified data gaps. Biogeosciences.

[5] IPCC. Summary for policymakers. In Stocker, T. F. *et al*. (eds.) *Climate Change 2013: The Physical Science Basis. Contribution of Working Group I to the Fifth Assessment Report of the Intergovernmental Panel on Climate Change* (Cambridge University Press, Cambridge, United Kingdom and New York, NY, USA, 2013).

[6] Schaphoff Sibyll, Heyder Ursula, Ostberg Sebastian, Gerten Dieter, Heinke Jens, Lucht Wolfgang (2013). Contribution of permafrost soils to the global carbon budget. Environmental Research Letters.

[7] Lawrence, D. M., Koven, C. D., Swenson, S. C., Riley, W. J. & Slater, A. G. Permafrost thaw and resulting soil moisture changes regulate projected high-latitude CO_2_ and CH_4_ emissions. *Environmental Research Letters* **10**, 094011, http://stacks.iop.org/1748-9326/10/i=9/a=094011 (2015).

[8] McGuire A. David, Lawrence David M., Koven Charles, Clein Joy S., Burke Eleanor, Chen Guangsheng, Jafarov Elchin, MacDougall Andrew H., Marchenko Sergey, Nicolsky Dmitry, Peng Shushi, Rinke Annette, Ciais Philippe, Gouttevin Isabelle, Hayes Daniel J., Ji Duoying, Krinner Gerhard, Moore John C., Romanovsky Vladimir, Schädel Christina, Schaefer Kevin, Schuur Edward A. G., Zhuang Qianlai (2018). Dependence of the evolution of carbon dynamics in the northern permafrost region on the trajectory of climate change. Proceedings of the National Academy of Sciences.

[9] Koven CD (2015). A simplified, data-constrained approach to estimate the permafrost carbon-climate feedback. Philosophical Transactions of the Royal Society A: Mathematical, Physical and Engineering Sciences.

[10] Gasser T (2018). Path-dependent reductions in co2 emission budgets caused by permafrost carbon release. Nature Geoscience.

[11] Koven CD, Lawrence DM, Riley WJ (2015). Permafrost carbon-climate feedback is sensitive to deep soil carbon decomposability but not deep soil nitrogen dynamics. Proceedings of the National Academy of Sciences.

[12] Beer Christian (2008). The Arctic carbon count. Nature Geoscience.

[13] Heimann M, Reichstein M (2008). Terrestrial ecosystem carbon dynamics and climate feedbacks. Nature.

[14] Schuur EAG (2015). Climate change and the permafrost carbon feedback. Nature.

[15] Zimov S, Zimov N, Tikhonov A, Chapin F (2012). Mammoth steppe: a high-productivity phenomenon. Quaternary Science Reviews.

[16] Olofsson, J. & Post, E. Effects of large herbivores on tundra vegetation in a changing climate, and implications for rewilding, **373**, 20170437 2018. 10.1098/rstb.2017.0437 PMC6231078 30348880

[17] Bernes C, Bråthen KA, Forbes BC, Speed JDM, Moen J (2015). What are the impacts of reindeer/caribou (Rangifer tarandus L.) on arctic and alpine vegetation? A systematic review. Environmental Evidence.

[18] Uboni A (2016). Long-term trends and role of climate in the population dynamics of eurasian reindeer. PLOS ONE.

[19] Zimov SA (2005). Pleistocene Park: Return of the Mammoth’s Ecosystem. Science.

[20] Ekici A (2014). Simulating high-latitude permafrost regions by the JSBACH terrestrial ecosystem model. Geosci. Model Dev..

[21] Porada P, Ekici A, Beer C (2016). Effects of bryophyte and lichen cover on permafrost soil temperature at large scale. The Cryosphere.

[22] Brovkin V (2013). Evaluation of vegetation cover and land-surface albedo in MPI-ESM CMIP5 simulations. Journal of Advances in Modeling Earth Systems.

[23] Ekici A (2015). Site-level model intercomparison of high latitude and high altitude soil thermal dynamics in tundra and barren landscapes. The Cryosphere.

[24] Chadburn SE (2017). Carbon stocks and fluxes in the high latitudes: using site-level data to evaluate Earth system models. Biogeosciences.

[25] Beer C, Porada P, Ekici A, Brakebusch M (2018). Effects of short-term variability of meteorological variables on soil temperature in permafrost regions. The Cryosphere.

[26] Webb, R. W., Rosenzweig, C. E. & Levine, E. R. Global soil texture and derived water-holding capacities (webb *et al*.), 10.3334/ORNLDAAC/548 (2000).

[27] Carvalhais N (2014). Global covariation of carbon turnover times with climate in terrestrial ecosystems. Nature.

[28] FAO/IIASA/ISRIC/ISSCAS/JRC. *Harmonized World Soil Database (version 1.2)* (FAO, Rome, Italy and IIASA, Laxenburg, Austria, 2012).

[29] Piani C (2010). Statistical bias correction of global simulated daily precipitation and temperature for the application of hydrological models. Journal of Hydrology.

[30] Beer C (2014). Harmonized European Long-Term Climate Data for Assessing the Effect of Changing Temporal Variability on Land-Atmosphere CO_2_ Fluxes. Journal of Climate.

[31] Giorgetta, M. *et al*. CMIP5 simulations of the Max Planck Institute for Meteorology (MPI-M) based on the MPI-ESM-LR model: The RCP85 experiment, served by ESGF 10.1594/WDCC/CMIP5.MXELr8 (2012).

[32] Brown, J., Ferrians, O., Heginbottom, J. A. & Melnikov, E. *Circum-Arctic Map of Permafrost and Ground-Ice Conditions, Version 2*, http://nsidc.org/data/ggd318 (Boulder, Colorado USA. NSIDC: National Snow and Ice Data Center, 2002).

[33] Christiansen HH (2010). he thermal state of permafrost in the nordic area during the international polar year 2007-2009. Permafrost and Periglacial Processes.

[34] Romanovsky V, Smith S, Christiansen H (2010). Permafrost thermal state in the polar northern hemisphere during the international polar year 2007–2009: A synthesis. Permafrost and Periglacial Processes.

[35] Smith S (2010). Thermal state of permafrost in north america: a contribution to the international polar year. Permafrost and Periglacial Processes.

[36] Raddatz T (2007). Will the tropical land biosphere dominate the climate–carbon cycle feedback during the twenty-first century?. Climate Dynamics.

[37] Reick CH, Raddatz T, Brovkin V, Gayler V (2013). Representation of natural and anthropogenic land cover change in MPI-ESM. Journal of Advances in Modeling Earth Systems.

[38] Verseghy DL (1991). Class-A Canadian land surface scheme for GCMS. I. Soil model. International Journal of Climatology.

[39] Weedon G (2011). Creation of the watch forcing data and its use to assess global and regional reference crop evaporation over land during the twentieth century. Journal of Hydrometeorology.

[40] Dee DP (2011). The ERA-Interim reanalysis: configuration and performance of the data assimilation system. Quarterly Journal of the Royal Meteorological Society.

[41] Meinshausen M (2011). The RCP greenhouse gas concentrations and their extensions from 1765 to 2300. Climatic Change.

[42] Hagemann S, Stacke T (2015). Impact of the soil hydrology scheme on simulated soil moisture memory. Climate Dynamics.

