# Supplementary material for: Protection of Permafrost Soils from Thawing by Increasing Herbivore Density
Source: Sci Rep. 2020 Mar 17;10:4170. doi: 10.1038/s41598-020-60938-y (PMC7078274; doi:10.1038/s41598-020-60938-y)
Supplement: Supplementary file 1 — Supplementary Information. [file 41598_2020_60938_MOESM1_ESM.pdf]

# Supplementary Information: Protection of Permafrost Soils from Thawing by Increasing Herbivore Density

**Christian Beer[1,2,3,*], Nikita Zimov[4], Johan Olofsson[5], Philipp Porada[1,2,6], and Sergey Zimov[4]**

[1]Department of Environmental Science and Analytical Chemistry, Stockholm University, Sweden
[2]Bolin Centre for Climate Research, Stockholm University, Sweden
[3]Institute of Soil Science, Department of Earth Sciences, Faculty of Mathematics, Informatics and Natural Sciences, Universität Hamburg, Germany
[4]North-East Scientific Station, Pacific Institute for Geography, Far-East Branch, Russian Academy of Sciences, Cherskii, Russia
[5]Department of Ecology and Environmental Sciences, Umeå University, Sweden
[6]Institute of Plant Science and Microbiology, Department Biology, Faculty of Mathematics, Informatics and Natural Sciences, Universität Hamburg, Germany
[*]christian.beer@uni-hamburg.de

## ABSTRACT

## Snow density evaluation

Snow density is a dynamic state variable in JSBACH. The model simulates snow densities of 200 to 260 kg m−3 in the permafrost region during DJF in 1990-2009 (Supplementary Information Fig. S1a). These estimates agree with an observed DJF mean of 210 kg m−3 (CI=[100 350]) for taiga/tundra ecosystems[1]. Our snow density estimates are at the lower end of the range reported by another recent modelling study[2]. JSBACH results agree with a spatial pattern of increasing snow density towards the north with lower tree cover[3] and colder temperatures[4].

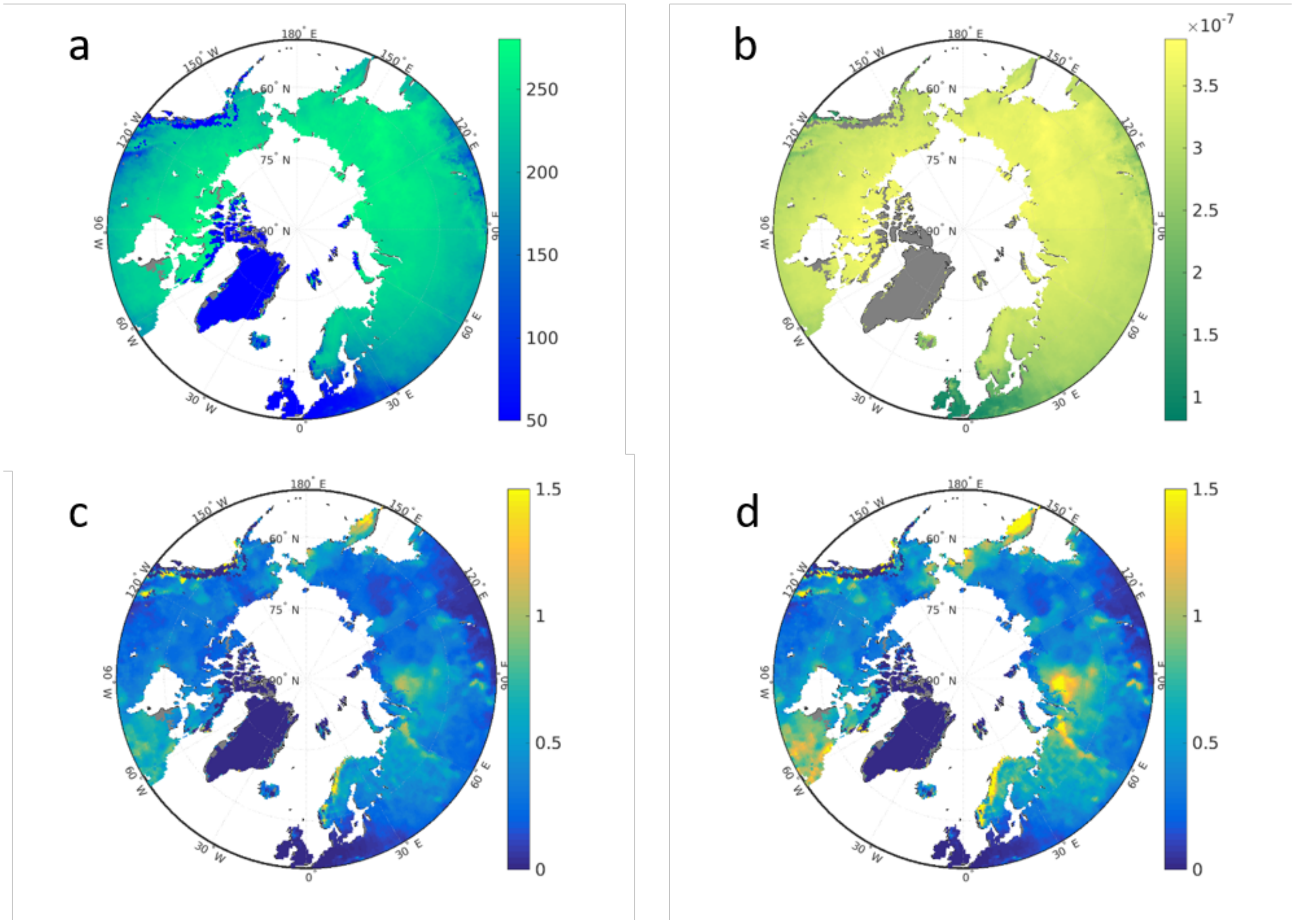


**Figure S1.** JSBACH decadal (1990-2009) snow properties. (a) DJF snow density (kg $m^{-3}$), (b) DJF snow thermal diffusivity ($m^{-2}$ $s^{-1}$), (c) DJF mean snow depth (m), and (d) annual maximum snow depth (m).

## Snow depth evaluation

The spatial pattern of snow depth follows general winter precipitation gradients, e.g. between East and West Canada and between West and East Siberia (Supplementary Information Fig. S1c,d). This pattern is similar to other global modelling studies[2,5]. The comparison to site-level observations from Canada[6] and Eurasia[7] shows a general validity of the model and climate forcing data with a bias in DJF snow depth of 7 cm and a root mean square error of 24 cm (Supplementary Information Fig. S2). This is in the range of biases from other modelling studies[5]. There is a trend of increasing positive bias towards mountain regions at the coast (Norway and Rocky Mountains) pointing to uncertainties in the winter precipitation forcing data applied. In Eurasia, model results agree better with observations in Central Europe and Siberia than in Scandinavia and Eastern Europe (Supplementary Information Fig. S2).

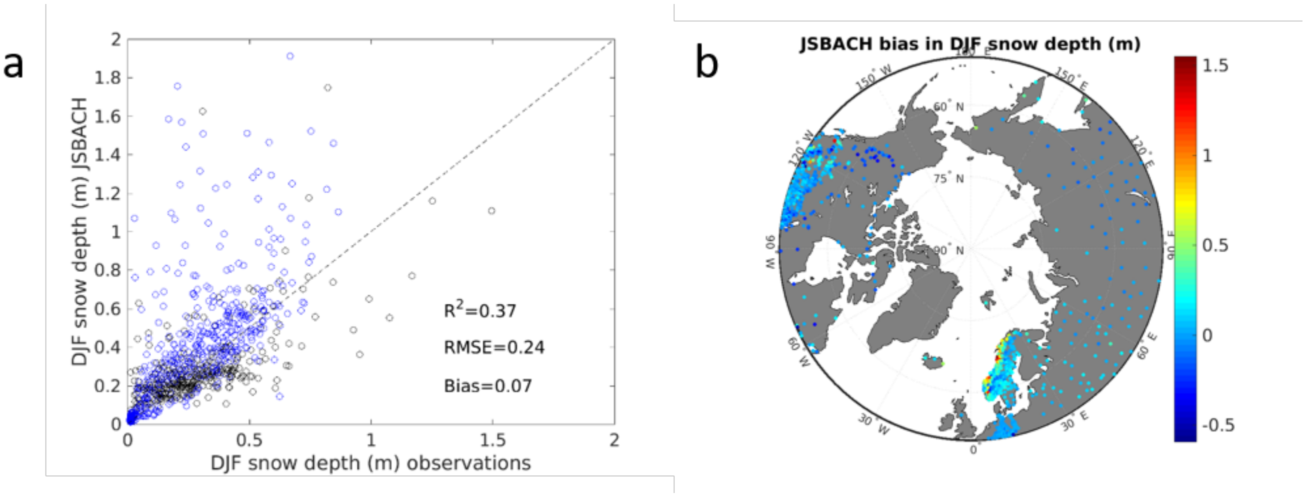


**Figure S2.** Evaluation of JSBACH decadal (1990-2009) DJF snow depth averages (m) against observations from Environment Canada (black) and European Climate Assessment (blue).

## Evaluation of snow insulation efficiency

Air temperature changes are reflected by soil temperature changes but there is a damping of the signal due to the insulation of soil by layers, such as snow, vegetation, litter etc. In northern high latitude regions snow is a particularly strong insulator. One possibility to evaluate the efficiency of this insulation is to compare observations and model results of a relationship between the soil-air temperature difference to snow depth. In Figure S3 this relationship is shown for two periods, early and late snow season, respectively. Observations are taken from[2]. In general, this comparison gives high confidence in the heat conduction scheme applied, the coefficient of determination is in both cases 0.77. The model underestimates snow insulation by 3 °C in the late snow season at snow depths less than 30 cm (Figure S3, red symbols) which is comparable to other modelling studies[2].

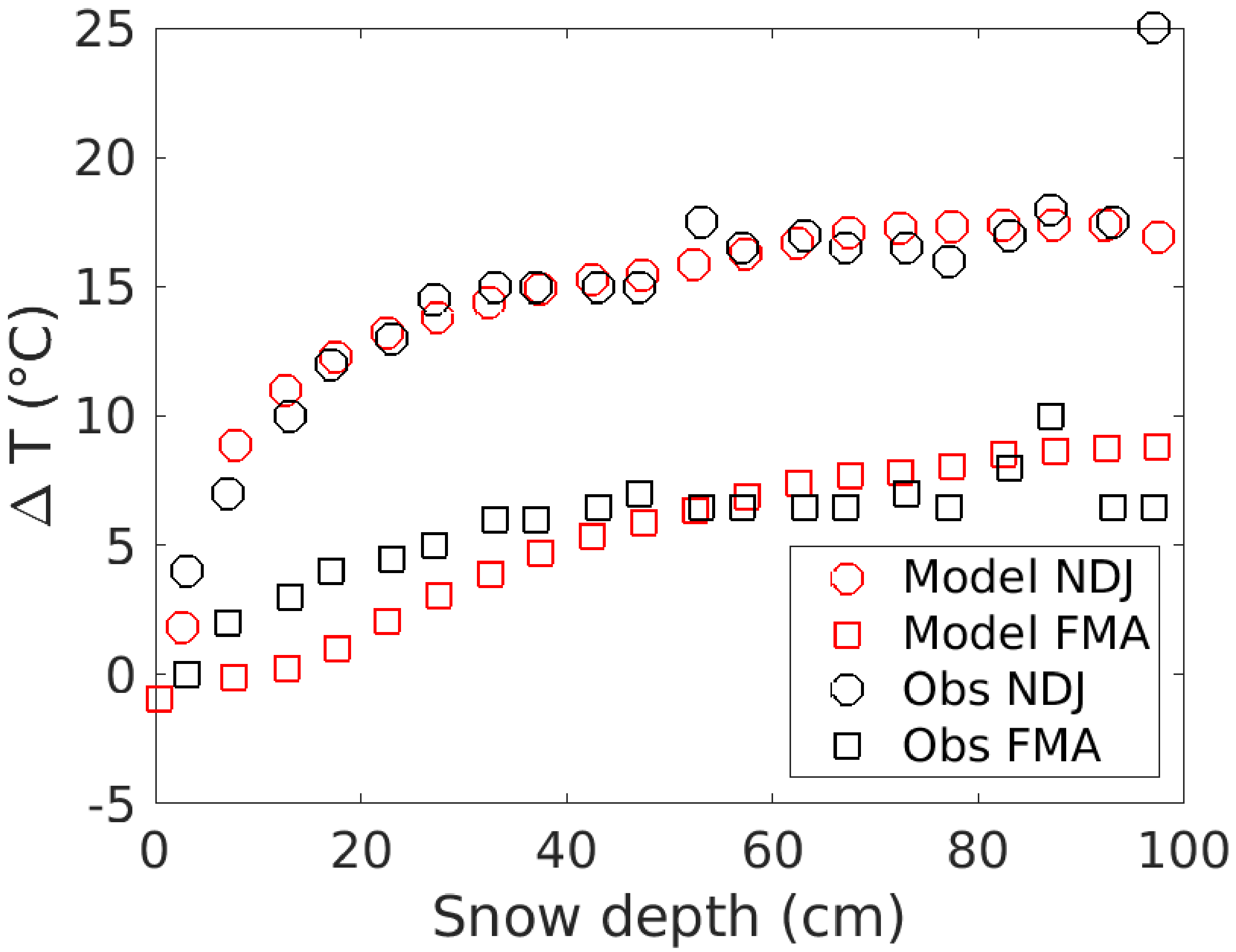


**Figure S3.** Evaluation of the efficiency of snow to insulate soil from air temperature. Shown is the relationship of the difference between soil temperature at 20 cm depth and air temperature (ΔT) to snow depth for two periods, November-December-January (NDJ) and February-March-April (FMA). CNTL model results are averaged over the entire permafrost region and during the period 1990-2009. Observations are taken from ref.[2] and represent the period 1981-2000.

## Land surface temperature evaluation

Model outputs during 2000-2009 are compared to a recent product for the Arctic which is based on satellite data[8] in Supplementary Information Fig. S4. JSBACH model results agree with the general pattern of land surface temperature in summer over the Arctic drainage basin. Differences are usually in the range of -2 to 2 °C . However, temperatures at the northern most boundary of the Siberian tundra are biased high with a difference between 2 and 6 °C . Another recent global modelling study shows a similar bias in that region[2].

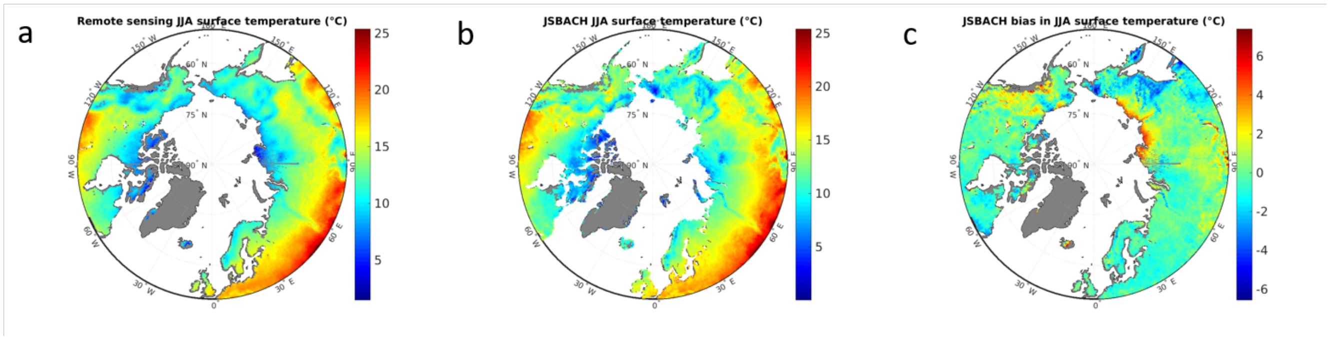


**Figure S4.** Evaluation of June-July-August land surface temperature (° C) during 2000-2009. (a) Satellite-derived[8] product. (b) JSBACH result. (c) Model result minus observation-based product.

## Mean annual ground temperature evaluation

This variable represents permafrost temperature in gelisols. It is similar (Supplementary Information Fig. S5) to borehole observations summarized for the International Polar Year by the GTN-P initiative[9–11]. There seems to be a cold bias in East Siberian mountains[12] but unfortunately only a few data points are available in that region for comparison (Supplementary Information Fig. S5). The small bias of -0.4 °C and the root mean square error of 2.4 °C confirms the applicability of the model at a pan-Arctic scale. For the whole northern high-latitude permafrost region, the model suggests a permafrost warming of 0.7 °C during the period 1980-2010. This change is in the range of 0.2 to 2 °C warming observed at several stations around the Arctic during the past decades[9–11]. This confirms that both spatial pattern and temporal dynamics of simulated mean annual ground temperature are valid for the investigation done in this study.

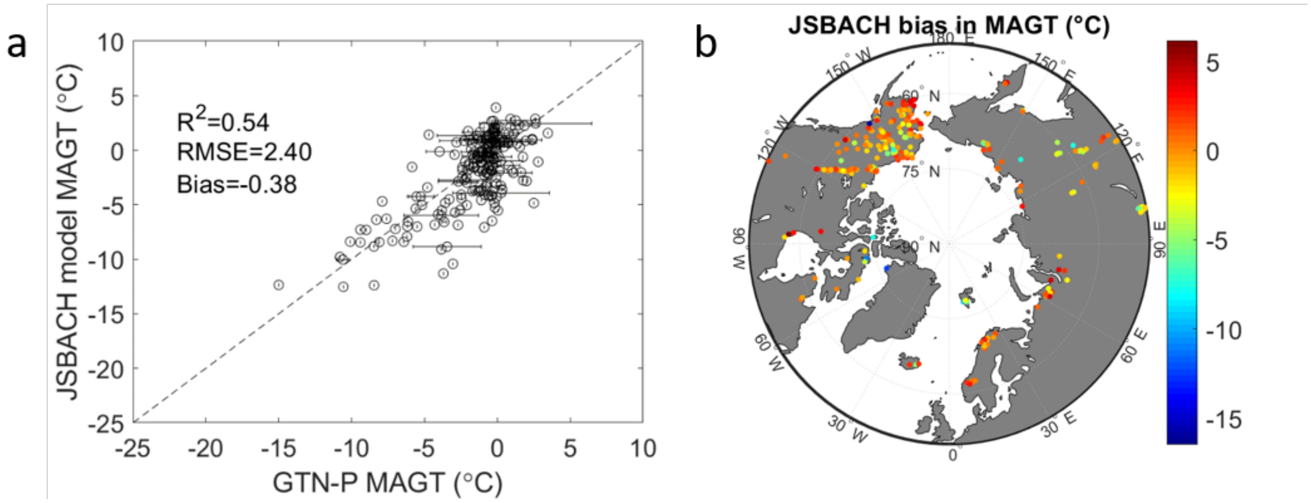


**Figure S5.** Evaluation of JSBACH permafrost temperature (°C ) against borehole measurements provided by the GTN-P initiative[9–11]. (a) Scatter plot and (b) spatial details of differences.

## Additional supplementary figures

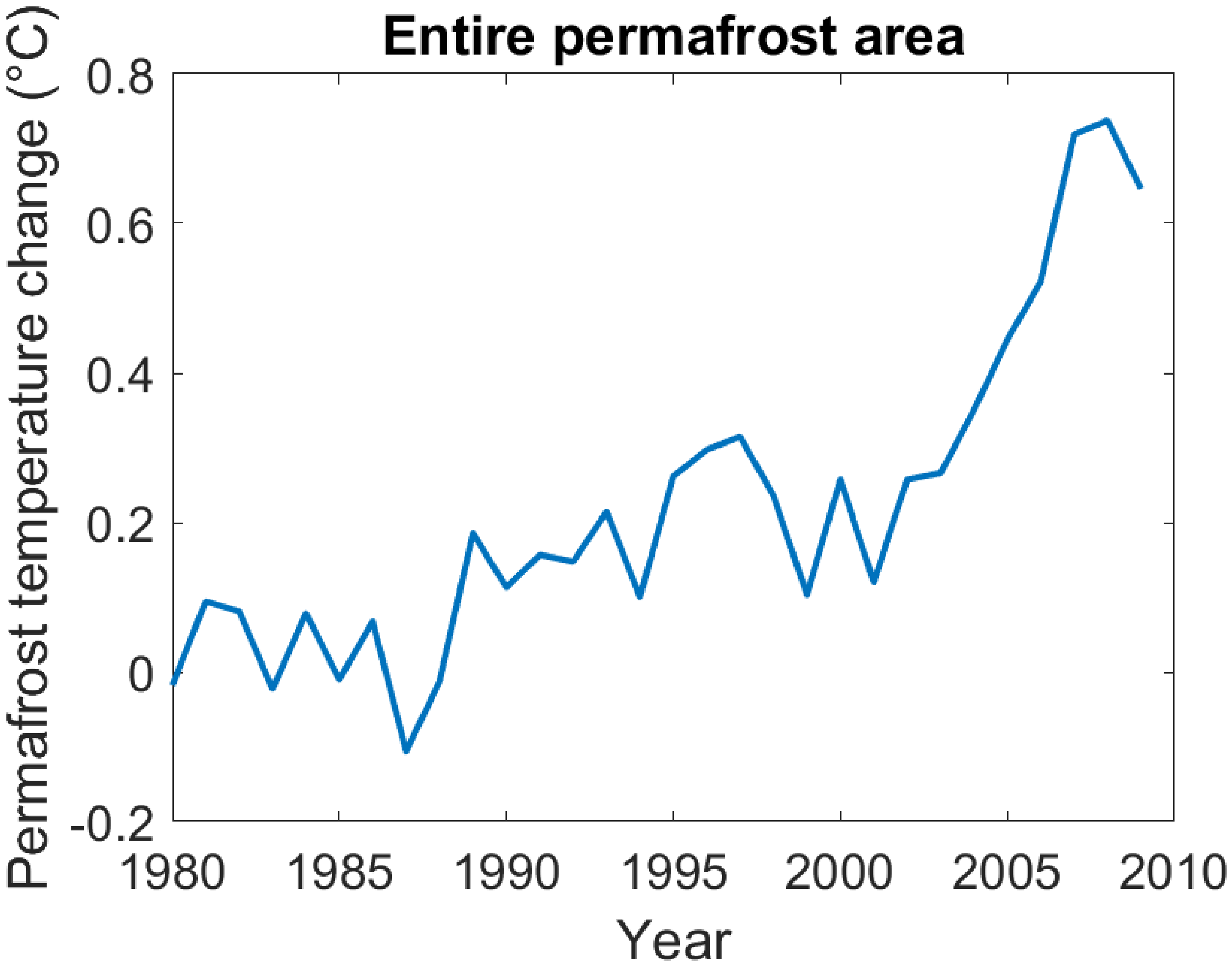


**Figure S6.** Permafrost mean annual ground temperature change (° C) averaged over the entire permafrost region. In comparison, observed warming ranges between 0.2 and 2 °C[9–11].

## References


1. Zhong, X., Zhang, T. & Wang, K. Snow density climatology across the former USSR. *The Cryosphere* **8**, 785–799, DOI: 10.5194/tc-8-785-2014 (2014).
2. Guimberteau, M. *et al.* ORCHIDEE-MICT (v8.4.1), a land surface model for the high latitudes: model description and validation. *Geosci. Model. Dev.* **11**, 121–163, DOI: 10.5194/gmd-11-121-2018 (2018).
3. Gouttevin, I. *et al.* How the insulating properties of snow affect soil carbon distribution in the continental pan-arctic area. *J. Geophys. Res. Biogeosciences* **117**, DOI: 10.1029/2011JG001916 (2012). G02020.
4. Hedstrom, N. R. & Pomeroy, J. W. Measurements and modelling of snow interception in the boreal forest. *Hydrol. Process.* **12**, 1611–1625, DOI: 10.1002/(SICI)1099-1085(199808/09)12:10/11<1611::AID-HYP684>3.0.CO;2-4 (1998).
5. Wang, W. *et al.* Evaluation of air–soil temperature relationships simulated by land surface models during winter across the permafrost region. *The Cryosphere* **10**, 1721–1737, DOI: 10.5194/tc-10-1721-2016 (2016).
6. Government of Canada. Monthly Climate Summaries, Historical Climate Data, Environment and Natural Resources, http://climate.weather.gc.ca/prods_servs/cdn_climate_summary_e.html (2018).
7. Klein Tank, A. M. G. *et al.* Daily dataset of 20th-century surface air temperature and precipitation series for the European Climate Assessment. *Int. J. Climatol.* **22**, 1441–1453, DOI: 10.1002/joc.773 (2002).
8. André, C., Ottlé, C., Royer, A. & Maignan, F. Land surface temperature retrieval over circumpolar Arctic using SSM/I-SSMIS and MODIS data. *Remote. Sens. Environ.* **162**, 1–10 (2015).
9. Christiansen, H. H. *et al.* The thermal state of permafrost in the nordic area during the international polar year 2007-2009. *Permafr. Periglac. Process.* **21**, 156–181 (2010).
10. Romanovsky, V., Smith, S. & Christiansen, H. Permafrost thermal state in the polar northern hemisphere during the international polar year 2007–2009: A synthesis. *Permafr. Periglac. Process.* **21**, 106–116 (2010).
11. Smith, S. *et al.* Thermal state of permafrost in north america: a contribution to the international polar year. *Permafr. Periglac. Process.* **21**, 117–135 (2010).
12. Beer, C., Porada, P., Ekici, A. & Brakebusch, M. Effects of short-term variability of meteorological variables on soil temperature in permafrost regions. *The Cryosphere* **12**, 741–757 (2018).
